# Water Hyacinth Leaves Are an Efficient, Green, and Cost-Effective Biosorbent for the Removal of Metanil Yellow from Aqueous Solution: Kinetics, Isotherm, and Thermodynamic Studies

**DOI:** 10.3390/molecules29143409

**Published:** 2024-07-20

**Authors:** Erick Aranda-García, Imelda Guerrero-Coronilla, Eliseo Cristiani-Urbina

**Affiliations:** Instituto Politécnico Nacional, Escuela Nacional de Ciencias Biológicas, Departamento de Ingeniería Bioquímica, Avenida Wilfrido Massieu s/n, Unidad Profesional Adolfo López Mateos, Alcaldía Gustavo A. Madero, Mexico City 07738, Mexico

**Keywords:** biosorption, metanil yellow, water hyacinth leaves, wastewater treatment

## Abstract

Excessive water hyacinth growth in aquatic environments and metanil yellow (MY) dye in industrial wastewater pose severe environmental and public health challenges. Therefore, this study evaluated the effects of various process factors on batch MY biosorption onto water hyacinth leaves (LECs) and MY biosorption kinetics, equilibrium, and thermodynamics. The optimal pH for MY biosorption by LECs was 1.5–2.0. The initial MY concentration affected the equilibrium MY biosorption capacity but not the LEC particle size and solution temperature. However, the LEC particle size and solution temperature affected the MY biosorption rate; the biosorption rate was higher at a lower particle size (0.15–0.3 mm) and a higher temperature (62 °C) than at higher particle sizes and lower temperatures. The pseudo-second-order model adequately described the biosorption kinetics of MY by LECs at the different levels of the process factors, whereas the Sips and Redlich–Peterson models satisfactorily represented the biosorption isotherm of MY. The Sips model predicted a maximum MY biosorption capacity of 170.8 mg g^−1^. The biosorption of MY by LECs was endothermic and not spontaneous. These findings demonstrate that LECs exhibit great potential for bioremediating MY-contaminated wastewater, thereby providing valuable insights for effective water treatment and pollution control strategies.

## 1. Introduction

Metanil yellow (MY; 3-(4-anilinophenylazo)benzene sulfonic acid sodium salt), also known as acid yellow 36 or tropaeolin [1,2], is a highly water-soluble anionic mono azo dye widely used in industries to color leather, nylon, wool, silk, varnish, ink, lacquer, paper, aluminum, detergents, soaps, plastics, cosmetics, pharmaceuticals, and pesticides [2,3,4,5]. Furthermore, owing to its low cost, high availability, and because it imparts an attractive golden yellow color to products, MY is also widely used as a coloring agent for ice creams, sweetmeats, beverages, and soft drinks, even though its use is banned in foods [3,4,5,6]. MY is also used as a pH indicator (pH range: 1.2–2.3) in potentiometric titrations [3]. However, MY has severe detrimental effects on ecosystems, humans, animals, and plants [2,5,7,8,9]. 

MY in aqueous environments affects the water transparency and natural water aesthetics, decreases the dissolved oxygen levels (thereby affecting the respiratory activity of aquatic organisms), affects the photosynthetic activities of aquatic plants because of reduced light penetration, and may be toxic to aquatic organisms because of its toxic breakdown products (e.g., p-amino diphenylamine) [1,2,6,10].

MY also affects human health and has diverse toxic effects on various human physiological systems, including the nervous, digestive, cardiovascular, excretory, and reproductive systems [4,11]. MY also generates oxidative stress and causes damage to all vital organs and organ systems in humans [4]. Furthermore, it causes skin allergy, weakness, giddiness, methemoglobinemia, cyanosis, and testicular lesions that decrease spermatogenesis and affect gene expression [2,3,6,12,13]. Additionally, MY is a potential mutagenic, carcinogenic, and genotoxic agent [3,5,6,7].

The negative environmental and human health impacts of MY exacerbate the need to effectively treat domestic and industrial wastewater containing the dye before discharge into aqueous environments. However, removing MY from wastewater using conventional physicochemical and biological methods is complicated because MY is highly soluble in water, highly stable in the environment, and resistant to degradation by common acids and bases, oxidizing chemical agents, oxygen, light, heat, and microorganisms owing to the complex molecular structure and synthetic nature of the dye [1,2,12]. Furthermore, these technologies have limitations such as high energy and reagent requirements, production of large amounts of sludge and uncontrollable products, high capital, operation and maintenance costs, low dye removal effectiveness and efficiency, low selectivity, and difficulty in adapting to a wide range of wastewater types [3,12,14,15,16]. Therefore, biosorption is a desirable alternative for treating dye-laden wastewater because of its effectiveness, efficiency, flexibility, ease of operation, cost-effectiveness, simple design, eco-friendliness, resistance to toxic contaminants, and biosorbent regeneration [17,18].

*Pontederia crassipes*, commonly known as water hyacinth and formerly known as *Eichhornia crassipes*, is a free-floating perennial hydrophyte that is one of the most invasive species of freshwater aquatic ecosystems in many countries on all continents [19,20,21]. *P. crassipes* reproduces rapidly, forming extensive and dense mats that blanket the water surface and prevent sunlight from reaching other aquatic plants, causing them to die. Therefore, water hyacinth affects the biodiversity of aquatic ecosystems because decay processes generate unpleasant odors, decrease water clarity, deplete dissolved oxygen in the water, and cause the death of aerobic aquatic fauna [22,23,24]. Water hyacinth in water bodies also interferes with shipping, navigation, irrigation, fishing, recreation and hydroelectric power generation activities and clog pipe systems for industry, agriculture, and municipal water supply [22,24,25]. Furthermore, these plants also create breeding areas for mosquitoes and other disease-causing vectors, increase evapotranspiration, and act as a channel for greenhouse gas emissions from water bodies [21,26,27,28]. 

Therefore, one of the most important concerns is how to take advantage of this highly available, high biomass yield (~110–120 t/ha/y), and low-cost aquatic plant [27] that is causing a negative impact on the environment, human health, fauna, flora, and economic development [29]. An economically, socially, and environmentally sound strategy is the sustainable use and valorization of water hyacinth as a renewable and green biosorbent for treating wastewater contaminated with recalcitrant toxic dyes, such as MY dye. Water hyacinth leaves (LECs) can biosorb MY from aqueous solutions. This capacity is more significant than that exhibited by the roots, stems, and the whole water hyacinth plant. Furthermore, the amide I and II functional groups of LEC proteins are primarily responsible for MY removal from aqueous solutions through electrostatic interactions [12]. Water hyacinth leaves (LECs) have also been successfully used for the batch [30] and continuous biosorption of acid red 27 (AR27) toxic dye [18] and reused in at least seven AR27 biosorption–desorption cycles in a batch system [31] and thirty AR27 biosorption–desorption cycles in a continuous system [32]. They have also been used effectively for the batch biosorption of alizarin yellow, rhodamine B [33], and Congo red [34] dyes, as well as of heavy metals, such as zinc, chromium [35], lead [36], cadmium [37], and copper [38,39,40].

However, the effects of physicochemical process factors on batch MY biosorption by LECs and MY biosorption kinetics, equilibrium, and thermodynamics are unclear, and the effectiveness of MY biosorption performance through LEC is unknown.

This study aimed to evaluate the effects of several physicochemical process factors (pH, initial MY concentration, temperature, contact time, and LEC particle size) on batch MY biosorption through LECs. Furthermore, the effectiveness of MY biosorption performance by LECs was assessed by modeling relevant data using kinetic, equilibrium, and thermodynamic models.

This study is the first to systematically investigate the biosorption of MY dye using water hyacinth leaves, providing a comprehensive analysis of the influence of process factors on MY biosorption, along with the biosorption kinetics, equilibrium, and thermodynamics involved.

## 2. Results and Discussion

### 2.1. Effect of pH

The solution pH is a critical factor in dye biosorption because it affects the solubility, dissociation, ionization extent, charge, and chemical structure of dye molecules [15,16,41,42]. The pH of the solution also influences the activity of the functional groups on the surface of the biosorbents, surface charge, chemical structure, and biosorbent properties. In addition, it affects the competition between dye molecules and coexisting ions in the solution by the biosorption active sites [15,16,19]. Consequently, the dye biosorption capacity, mechanism, and process efficiency strongly depend on the solution pH [42].

**Figure 1:**
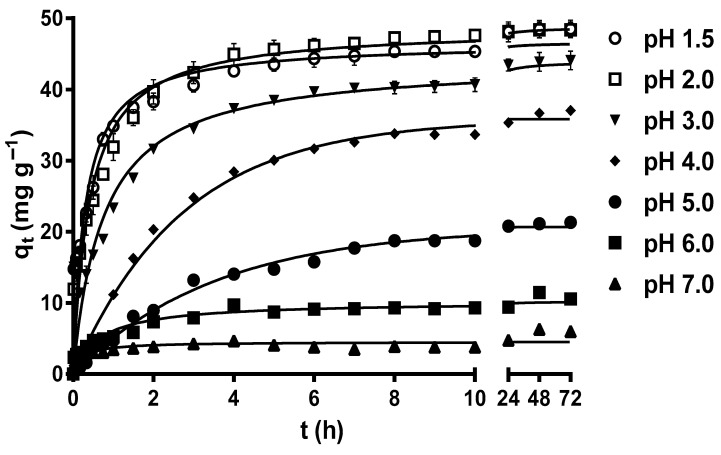
Effect of the solution pH on metanil yellow (MY) biosorption by water hyacinth leaves (LECs). Conditions: Initial MY concentration = 50 mg L^−1^; LEC concentration = 1 g L^−1^; LEC particle size = 0.15–0.3 mm; solution temperature = 21 ± 1 °C. (—, pseudo-second-order kinetic model prediction).

Figure 1 displays the variation profiles of the MY biosorption capacity of LEC with respect to time at different pH levels. After 72 h, the MY biosorption capacity increased from 4.21 to 45.65–45.80 mg g^−1^ as the solution pH decreased from 7 to 1.5–2.0, without a significant difference (*p* > 0.05) for these last two pH values (pH 1.5 and 2.0). These results indicate that the optimal pH values for MY biosorption by LECs are 1.5 and 2.

The strong influence of the solution pH on MY biosorption by LECs can be explained by the LEC zero-charge point pH (pH_pzc_) and the LEC surface zeta potential (ζ) values at different solution pH levels. The LEC pH_pzc_ is 2.37, and that of the LEC ζ values are positive when the solution pH < pH_pzc_ and negative when the solution pH > pH_pzc_. Furthermore, the LEC ζ values become more negative as the solution pH increases from pH_pzc_ = 2.37 to pH 10 [18]. These results indicate that the LEC surface has a net positive electrical charge when the solution pH < pH_pzc_, a net negative electrical charge when the solution pH > pH_pzc_, and that the net electrical charge of the LEC surface becomes more negative as the solution pH increases from pH_pzc_ = 2.37 to pH 10. Therefore, the highest values of MY biosorption capacity in this study when solution pH 1.5 and 2.0 are caused by the large electrostatic attraction forces between the positive charges of the LEC surface and the negative charges of the anionic MY dye [17,18,30]. These findings are consistent with those of Guerrero-Coronilla et al. [12], who reported that MY biosorption from acidic solutions by LECs is caused by the electrostatic attraction between the negatively charged sulfonic groups of MY and the positively charged amide groups of LEC proteins. In contrast, the decrease in the MY biosorption capacity as the solution pH increased from 3.0 to 7.0 is attributed to the fact that the repulsive electrostatic forces between the negative charges of the LEC surface and that of the anionic MY dye increased with increase in the solution pH [30,43].

In this study, the optimal solution pH values for MY biosorption by LECs were 1.5 and 2.0, with a MY equilibrium biosorption capacity of approximately 45.8 mg g^−1^. Based on these results, subsequent studies were performed at a solution pH of 2.0. Similarly, solution pH values in the range of 2–7.9 are optimal for MY biosorption by adsorbents/biosorbents (Table 1) [1,7,8,13,15,43,44,45,46,47,48,49], with pH 2.0 being optimal for MY biosorption by de-oiled soybean and bottom ash [46].

### 2.2. Effect of LEC Particle Size

**Figure 2:**
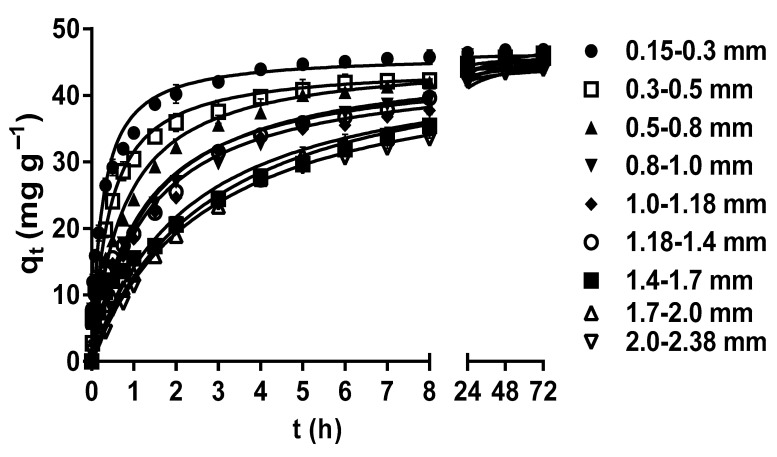
Effect of the LEC particle size on MY biosorption. Conditions: Initial MY concentration = 50 mg L^−1^; solution pH = 2.0; LEC concentration = 1 g L^−1^; solution temperature = 21 ± 1 °C. (—, pseudo-second-order kinetic model prediction).

The particle size plays a crucial role in determining the specific surface area of the biosorbent, as well as the accessibility and availability of biosorption active sites on its surface [42,50,51]. The effects of LEC particle size on the MY biosorption capacity are shown in Figure 2. The MY biosorption capacity and rate increased as the LEC particle size decreased during the first 8 h of contact between the LEC biosorbent and MY solution. This increase may be attributed to the facts that, when the biosorbent particle size is reduced, the contact surface area between the biosorbent and the liquid phase increases, the diffusion of dye molecules into inner surface sites is shortened, the intraparticle diffusion resistance is reduced, and the accessibility to internal biosorbent binding sites is improved. Consequently, the biosorption capacity and rate increase, and the time to reach dynamic equilibrium is reduced [15,52].

However, after 8 h, all LEC particle sizes had the same MY biosorption capacity in such a way that no significant difference in the MY equilibrium biosorption capacity (45.66 mg g^−1^) by the different particle sizes (*p* > 0.05) was observed at 48–72 h. This behavior is expected in porous materials, such as LECs, whose external surface area is negligible compared to their internal surface area. Hence, the contribution of the external surface area to the total surface area is limited. Therefore, the reduced particle size of porous materials does not significantly affect the total surface area and the equilibrium biosorption capacity [30,53]. In contrast, the particle size affected the MY biosorption rate because the time required to reach equilibrium decreased with the decrease in the particle size, from 24 h for a particle size of 2.0–2.38 mm to 5 h for a particle size of 0.15–0.3 mm (Figure 2). As the MY biosorption rate increased with the decrease in the LEC particle size, the time required to reach maximum biosorption capacity was shorter with the 0.15–0.3 mm particle size; therefore, this particle size was used in further studies.

These results indicate that the LEC particle size affected the biosorption rate but not the MY equilibrium biosorption capacity. A similar behavior was observed in the biosorption studies of acid red 27 dye by LECs [30], acid orange 7 dye by *Stoechospermum marginatum* algae biomass [50], and chromium(III) by orange waste [53]. 

### 2.3. Influence of Initial MY Concentration and Contact Time

The contact time between a biosorbent and an adsorbate solution is crucial in batch biosorption processes. It determines the duration (operation time) of biosorption and provides essential information on the biosorption kinetics of a biosorbent for a specific adsorbate and biosorbent concentrations [54,55]. Furthermore, the time required to reach the dynamic equilibrium state or equilibrium time, offers valuable insights into the efficiency of a biosorbent in biosorbing a particular adsorbate. This information is key to assessing the potential success of the biosorbent in practical applications [56].

**Figure 3:**
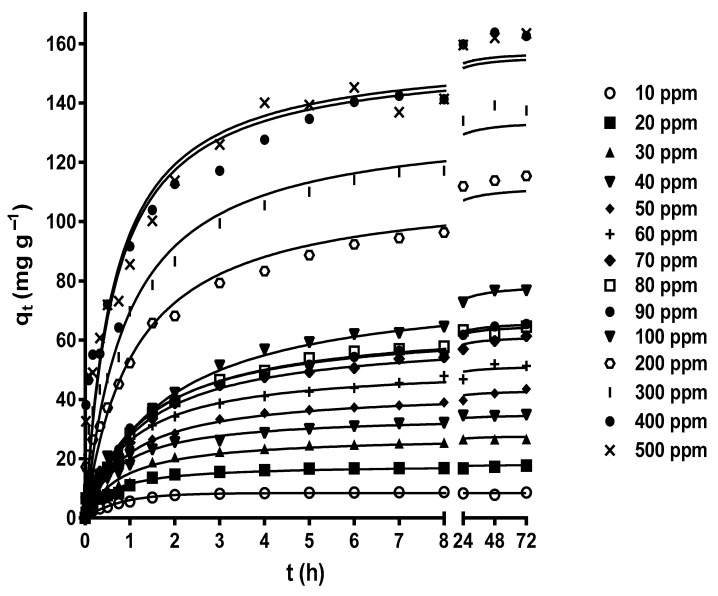
Influence of the contact time and initial MY concentration on MY biosorption. Conditions: Solution pH = 2.0; LEC concentration = 1 g L^−1^; LEC particle size = 0.15–0.3 mm; solution temperature = 21 ± 1 °C. (—, pseudo-second-order kinetic model prediction).

Figure 3 shows the effect of contact time on MY biosorption by LEC when initial MY concentrations of 10–500 mg L^−1^ were used. The contact time strongly affected the MY biosorption at all initial MY concentrations. The MY biosorption capacity increased rapidly during the initial stage of biosorption, then increased slowly as the experimental biosorption time increased, until it gradually reached a plateau with a constant maximum biosorption capacity value corresponding to the equilibrium biosorption capacity. After this point, MY biosorption from the aqueous solution was no longer detected. The results in Figure 3 agree with the experimental findings of MY adsorption by glutaraldehyde cross-linked magnetic chitosan nanoparticles [44], alginate-immobilized aquatic weed [45], spent *Rhizopus arrhizus* biomass [43], bottom ash, and de-oiled soybean [46].

The high MY biosorption rate in the initial stage of biosorption was possibly caused by the many active binding sites available for biosorption and a high concentration gradient driving force to transfer the MY molecules from the bulk of the liquid phase to the LEC surface [56]. The subsequent progressive decrease in the MY biosorption rate resulted from the decreased vacant binding sites and MY concentration gradient driving force until the net biosorption rate became zero and the dynamic equilibrium state was reached.

The initial adsorbate concentration is another crucial factor that affects biosorption because it is the driving force for adsorbate diffusion and mass transfer to take place from the bulk of the aqueous solution to the biosorbent surface [15,42,56]. 

In this study, various initial MY concentrations (10–500 mg L^−1^) were assayed, thereby considering the possible concentrations of dyes in industrial liquid effluents. The MY equilibrium biosorption capacity increased as the initial MY concentration increased, from 6.285 mg g^−1^ for a MY concentration of 10 mg L^−1^ to 163.5 mg g^−1^ for a MY concentration of 500 mg L^−1^ (Figure 3). These results are attributed to the increased initial MY concentration, which favored interactions between LEC and MY molecules owing to the greater availability of MY molecules in the aqueous solution. Additionally, the increased concentration gradient driving force of MY helped to overcome the mass transfer resistances of MY molecules from the bulk of the aqueous solution to the LEC surface. This led to the saturation of the active biosorption sites on the outer and inner surfaces of LECs and an increase in the MY biosorption capacity [57,58]. These results indicate that the initial MY concentration is a crucial factor that affects the saturation of the LEC surface with MY molecules.

### 2.4. Effect of Temperature

Although the solution temperature affects the biosorption of dyes to a lesser extent than other factors, such as the solution pH [51], the temperature has a positive effect on dye biosorption [59]. The solution temperature can affect the solubility, surface activity, and diffusion rate of dyes, as well as the physical and chemical structure, biosorption active sites, and biosorbent activity [42,51,59]. 

**Figure 4:**
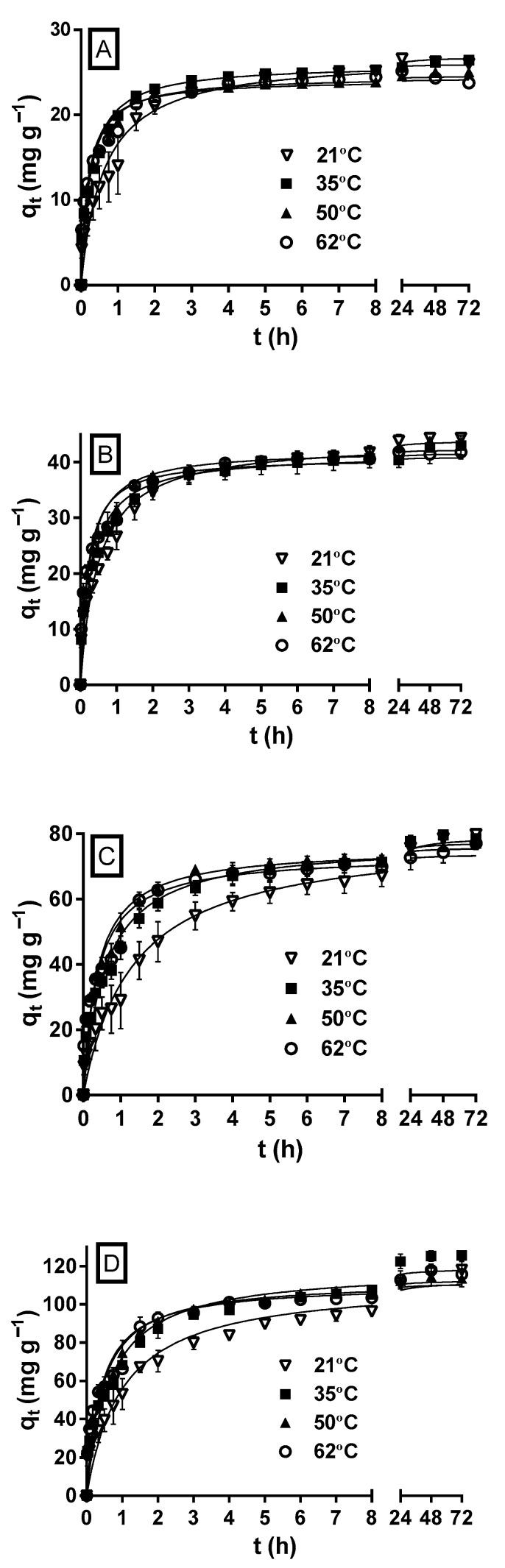
Effect of the solution temperature on MY biosorption by LECs. Conditions: Solution pH = 2.0; LEC concentration = 1 g L^−1^; LEC particle size = 0.15–0.3 mm; Initial MY concentration: (**A**) 30; (**B**) 50; (**C**) 100; (**D**) 200 mg L^−1^. (—, pseudo-second-order kinetic model prediction).

Figure 4 shows the variation profiles of MY biosorption capacity as a function of the experimentation time at the different initial MY concentrations and solution temperatures. At all the initial MY concentrations, but primarily at the highest initial concentrations (100 and 200 mg L^−1^) assayed, the MY biosorption capacity and rate increased during the first hours as the solution temperature increased; therefore, the equilibrium state was reached faster at higher temperatures than at lower temperatures.

These results indicate that MY biosorption by LECs is endothermic and attributed to the fact that increasing the solution temperature increases the surface activity and diffusion rate of MY molecules, decreases the boundary layer thickness that surrounds the LEC particles (which decreases the mass transfer resistances in the boundary layer), increases the LEC surface activity, and favors the interactions between dye molecules and LEC biosorption active sites [30].

However, the biosorption capacities and rates approached the same value as the biosorption time progressed for a given initial MY concentration and at all the solution temperatures. The differences in the MY biosorption capacities were negligible when equilibrium was reached. These results indicate that the solution temperature affected the rate but not the equilibrium capacity of MY biosorption. In contrast, increasing the temperature increases the MY equilibrium biosorption capacity of pitaya peel [1] and de-oiled soybean but decreases that of bottom ash [46]. Furthermore, at all the initial MY concentrations, the influence of the solution temperature on the MY equilibrium biosorption capacity of LECs was small compared to that caused by other factors, such as the solution pH, LEC particle size, and initial MY concentration. These results concur with previous studies that did not observe a remarkable change in the equilibrium biosorption capacity with the change in the solution temperature [30,60].

### 2.5. MY Biosorption Kinetics Modeling

The modeling of biosorption kinetic processes provides essential information on the performance of the biosorbent used, the response of a biosorption system to changes in environmental factors, biosorbent properties, biosorption rate, as well as the steps and factors that control the biosorption rate and mechanism [56,61]. Therefore, information on biosorption kinetics can be used to design and optimize effective biosorption systems [57].

The pseudo-first-order, pseudo-second-order, Elovich, and fractional power kinetic models were used to model the kinetic process of MY biosorption onto LECs at solution pH levels from 1.5 to 7.0, LEC particle sizes from 0.15–0.3 to 2.0–2.38 mm, initial MY concentrations from 10 to 500 mg L^−1^, and temperatures from 21 to 62 °C. Tables S1–S4 (Supplementary Material) display the experimental MY biosorption capacity at equilibrium (q_e_exp_), the values of the parameters of the kinetic models, and the corresponding determination coefficient (R^2^), sum of squares error (SSE), and root-mean-squared error (RMSE) values for MY biosorption at the different operation process conditions.

Generally, the pseudo-second-order model yielded the highest R^2^ values, the lowest SSE and RMSE values, and the narrowest confidence intervals at the different pH levels, biosorbent particle sizes, initial MY concentrations, and temperatures assayed. Furthermore, the biosorption capacities predicted by the pseudo-second-order model were close to those obtained experimentally. These results indicate that the pseudo-second-order model is the most suitable for describing the kinetic process of MY biosorption by LECs.

These results agree with previous studies [12] that found that the pseudo-second-order model best fits the kinetic profile of MY biosorption by the leaves, stems, roots, and entire water hyacinth plant at different contact times. Furthermore, the present results also agree with that reported by Madikizela [19], who stated that the pseudo-second-order model most appropriately represents the kinetics of biosorptive removal of organic contaminants by water hyacinth. The pseudo-second-order model assumes that the biosorption rate of the adsorbate on the biosorption binding sites is directly proportional to the square of the number of binding sites available on the biosorbent’s surface. The pseudo-second-order model has adequately described the MY biosorption by multiple biosorbents and adsorbents (Table 1). The high fit of experimental data to the pseudo-second-order model suggests that chemisorption is the rate-controlling step in MY biosorption process onto LECs [12,15]. Chemisorption primarily comprises electrostatic interactions, ion exchange, the formation of complexes between the functional groups of the biosorbent with adsorbate, and precipitation [16].

The highest values of the rate constant (k_2_) of the pseudo-second-order model were obtained at solution pH values of 1.5 and 2.0, in which the highest MY biosorption capacities were reached without significant differences from each other. This study also revealed a correlation between the rate constant k_2_ and the LEC particle size. As the particle size decreased from 2.0–2.38 to 0.15–0.30 mm, the rate constant k_2_ increased in the range of 0.008–0.089 g mg^−1^·h^−1^. This increase indicates that the MY biosorption rate is faster with smaller particle sizes, leading to a quicker attainment of equilibrium. Another key finding is the inverse relationship between the rate constant k_2_ of the pseudo-second-order model and the initial MY concentration. As the concentration increased, the rate constant k_2_ decreased. This result can be attributed to the fact that LEC biosorbed more MY molecules on its surface at higher initial MY concentrations than at lower MY concentrations, leading to a longer time required to reach equilibrium. This finding is consistent with observations in other biosorption systems [30]. In addition, the rate constant k_2_ increased with the increase in the temperature at all the initial MY concentrations, possibly because, as the temperature increased, the interactions between LEC and the MY dye also increased. Therefore, the biosorption rate is faster at high temperatures than at low temperatures; these results also indicate the endothermic nature of MY biosorption by LECs [30].

### 2.6. MY Isotherm and Its Modeling

Elucidating the adsorbate biosorption isotherm, which describes and predicts the biosorption capacity as a function of the equilibrium adsorbate concentration, is crucial for designing and optimizing biosorption processes at an industrial level [43,56,62,63]. Sorption isotherms and the mathematical models that describe them can characterize the biosorption process in a complete and detailed manner [62].

**Figure 5:**
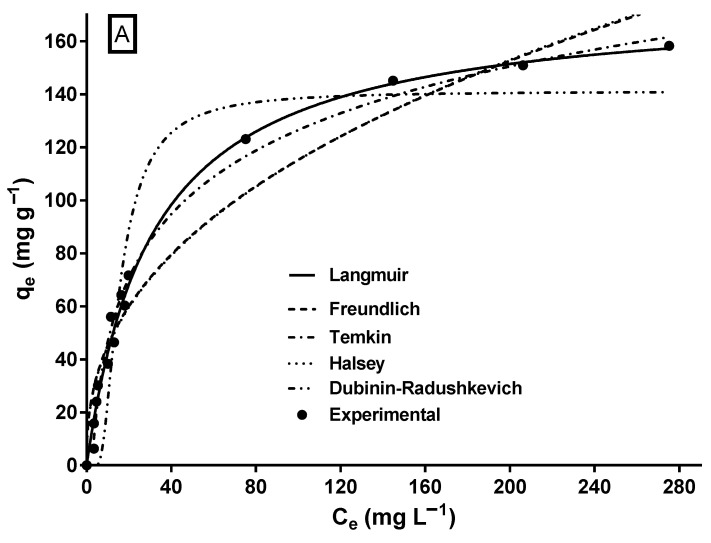

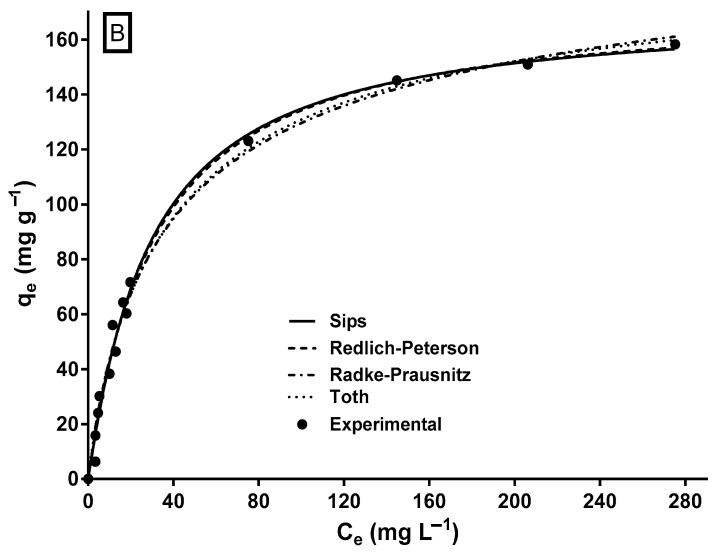
Experimental and model-predicted isotherm data for MY biosorption onto LECs. (**A**) Two-parameter isotherm models and (**B**) three-parameter isotherm models.

The biosorption isotherm of MY on LECs is shown in Figure 5. The shape of the isotherm corresponds to an L-type isotherm of Giles’ classification [64], which is typical of biosorbents with a high affinity for adsorbates [65] and when there is no strong competition between the adsorbate molecules and the solvent [30].

A characteristic of the L-type isotherm is that the slope of the isotherm gradually decreases as the adsorbate concentration increases, suggesting that the vacant biosorption sites decrease as the surface of the biosorbent is covered with the adsorbate [66], resulting in progressive saturation of the biosorbent [67]. Likewise, the L-type isotherm reflects the biosorption of an adsorbate monolayer and chemisorption [67], which is consistent with the results of the MY biosorption kinetic study.

The mathematical modeling of biosorption isotherms provides information on the mechanisms involved in biosorption to predict the maximum biosorption capacity, elucidate the interactions and affinity between the adsorbate and the biosorbent, design the biosorption process, and optimize the usage of the biosorbent in large-scale biosorption systems. Therefore, determining the most suitable isotherm model for the biosorption system is essential. In this study, two-parameter (Langmuir, Freundlich, Halsey, Temkin, and Dubinin–Radushkevich) and three-parameter (Sips, Redlich–Peterson, Radke–Prausnitz, and Toth) isotherm models were used to model the experimental data of the equilibrium biosorption of MY on LECs. The parameter estimates and error statistics of the isotherm models are shown in Table 2, and the profiles predicted by the two- and three-parameter models, respectively, are presented in Figure 5A,B.

Except for the Dubinin–Radushkevich model, all assayed isotherm models yielded an R^2^ ≥ 0.95 and relatively low SSE and RMSE values. The Sips and Redlich–Peterson models showed the best fit to the experimental equilibrium data of MY biosorption onto LECs, according to their highest R^2^ (0.992) values and their lowest RMSE and SSE values. The Sips and Redlich–Peterson models are hybrid models that result from combining the Langmuir and Freundlich isotherm models and are used for homogeneous and heterogeneous adsorption systems [62,63]. In the present study, the exponents of the Sips (n_s_ = 1.058) and Redlich–Peterson (β_RP_ = 1.012) models are close to the unity. In these cases (when n_s_ = 1.0 and β_RP_ = 1.0), the Sips and Redlich–Peterson models become the Langmuir model; therefore, the Langmuir model is also suitable to represent the equilibrium adsorption of MY onto LECs (Figure 5A) and in the error statistics (R^2^, RMSE, and SSE in Table 2). The Langmuir model is the best isotherm model to represent the equilibrium biosorption of MY onto several adsorbents/biosorbents (Table 1).

The q_max_ values are an essential parameter to compare the capacity of different adsorbents/biosorbents used to remove MY dye. The maximum MY biosorption capacity predicted by the Sips model (q_maxS_) was 170.8 mg g^−1^ (Table 2), close to that achieved experimentally (163.5 mg g^−1^). The maximum MY biosorption capacity of LECs is greater than that of most of the listed biomaterials (Table 1). Therefore, LECs are a strong MY biosorbent. Furthermore, the maximum MY biosorption capacity predicted by the Langmuir model for a few of the adsorbents listed in Table 1 is higher than those achieved by LECs. Notably, some predicted q_max_ values shown in Table 1 are much higher than those achieved experimentally. Furthermore, conventional and synthetic adsorbents, such as activated carbon, zeolites, ion exchange resins, silica, bentonite, alumina, and their derivatives, usually cost more than biosorbents [68].

### 2.7. Thermodynamics of MY Biosorption

Determining the primary thermodynamic parameters of MY biosorption onto LECs, including activation energy, and changes in activation enthalpy, entropy, and Gibbs free energy, significantly contributes to understanding this process. These parameters were derived using the Arrhenius, Eyring–Polanyi, and Gibbs equations and the rate constants of the pseudo-second-order (k_2_) model obtained in the kinetic study at different temperatures. This kinetic model was selected because it best fits the experimental data of MY biosorption onto LECs, further validating the importance of these findings.

As the initial dye concentration increased, both parameters (activation energy (E_A_) and frequency factor constant (A_o_)) decreased, indicating a correlation between the minimum energy required for the biosorption reaction of MY on LECs and the initial dye concentration (Table 3). The E_A_ values, ranging from 16.50 to 24.30 kJ mol^−1^, fell within the E_A_ range values of 8.4–83.7 kJ mol^−1^ for chemisorption processes. These findings suggest that MY biosorption onto LECs is primarily driven by chemical sorption reactions, which aligns with the kinetic and equilibrium study results.

The changes in activation enthalpy and activation entropy were obtained from the Eyring–Polanyi equation, with positive and negative values at all the initial concentrations of MY, respectively. The positive values of the activation enthalpy change confirm the endothermic nature of MY biosorption onto LECs. In contrast, negative and relatively low values of the activation entropy change indicate a decreased randomness at the solid–liquid interface after biosorption, stable chemical complex formation on the surface of the LEC, and that minor changes occur in the internal structure of LECs during biosorption [46,50,69,70].

The Gibbs activation free energy change was positive at all temperatures and initial MY concentrations, which indicates that MY biosorption onto LECs is not spontaneous. This finding suggests the presence of an energy barrier for dye biosorption, necessitating an energy supply to overcome the barrier for the biosorption reaction to occur. This result aligns with those of other studies [56,69]. Table 1 further illustrates the energetic (endothermic or exothermic) and spontaneity (spontaneous or non-spontaneous) nature of various MY adsorption/biosorption systems, highlighting the diversity of these systems.

Our findings offer practical implications for improving water treatment methodologies and mitigating pollution.

## 3. Materials and Methods

### 3.1. Biosorbent

Water hyacinth (*P. crassipes*) plants were collected from the Xochimilco canals (19°15′30.7″ N 99°05′00.3″ W) in the San Gregorio Atlapulco area, Mexico City, Mexico. The leaves were cut at the base of the petiole, thoroughly washed with tap water to remove adhering particles and water-soluble impurities, and washed with distilled water. The leaves were then cut into small pieces, dried at 60 °C for 48 h, ground in a Glen Creston hammer mill (Glen Creston Ltd., London, UK), and sieved using ASTM standard sieves to obtain fractions with different particle sizes (0.15–0.3, 0.3–0.5, 0.5–0.8, 0.8–1.0, 1.0–1.18, 1.18–1.4, 1.4–1.7, 1.7–2.0, and 2.0–2.38 mm). The sieved fractions were stored separately in tightly closed glass bottles at room temperature (21 °C).

### 3.2. MY Stock and Test Solutions

A stock solution of MY dye (purity ≥ 98%; Sigma-Aldrich, St. Louis, MO, USA) of 1 g L^−1^ was prepared. The test solutions were prepared by diluting the MY stock solution with distilled water, and the pH was adjusted to the desired values by adding 0.1 M HCl or 0.1 M NaOH (JT Baker, Avantor Performance Materials, Xalostoc, Estado de México, Mexico).

### 3.3. Kinetic Studies of the Effects of Several Batch Processing Factors on MY Biosorption

Biosorption kinetic studies were performed to determine the effects of solution pH, LEC particle size, contact time between the biosorbent and MY solution, initial MY concentration, and temperature upon batch MY biosorption from aqueous solutions by LEC. The biosorption experiments were performed in 500 mL Erlenmeyer flasks containing 125 mL of MY solution of known pH and dye concentration and a biosorbent concentration of 1 g L^−1^. The pH of each test solution was kept constant at the desired value over the entire period of contact time between the biosorbent and the MY solution by adding 0.1 M HCl or 0.1 M NaOH. The flasks were kept under constant shaking (140 rpm) on an orbital shaker (Cole Palmer, Vernon Hills, IL, USA) for 72 h.

The effect of pH on the kinetic performance of MY biosorption was investigated by varying the solution pH in the range of 1.5–7 (1.5, 2.0, 3.0, 4.0, 5.0, 6.0, and 7.0), while the initial concentration of MY, LEC particle size, and solution temperature were 50 mg L^−1^, 0.15–0.3 mm, and 21 ± 1 °C, respectively. The solution pH at which the highest biosorption capacity was reached was used in subsequent experiments. A dye solution with an initial MY concentration of 50 mg L^−1^ and a temperature of 21 ± 1 °C was used, and the LEC particle size was varied in the range of 0.15–2.38 mm (0.15–0.30, 0.30–0.50, 0.50–0.80, 0.80–1.0, 1.0–1.18, 1.18–1.40, 1.40–1.70, 1.70–2.0, and 2.0–2.38 mm) to evaluate the influence of the LEC particle size on MY biosorption. The particle size range with the best MY biosorption characteristics was selected for the subsequent experiments. The effect of contact time on MY biosorption was examined for 0, 0.02, 0.08, 0.17, 0.33, 0.50, 0.75, 1.0, 1.5, 2.0, 3.0, 4.0, 5.0, 6.0, 7.0, 8.0, 24, 48, and 72 h in solutions with initial MY concentrations of 10, 20, 30, 40, 50, 60, 70, 80, 90, 100, 200, 300, 400, and 500 mg L^−1^ at 21 ± 1 °C. These initial MY concentrations were also used to assess the influence of dye concentration on its biosorption on LECs, and the temperature of the solutions was maintained at 21 ± 1 °C. The influence of temperature on MY biosorption was investigated by varying the temperature from 21 to 62 °C (21, 35, 50, and 62 ± 1 °C), and four initial MY concentrations were used (30, 50, 100, and 200 mg L^−1^).

Simultaneously and under the same operating conditions at which the biosorption experiments were performed, LEC-free controls were run to detect any possible MY removal due to photodegradation, precipitation, or adsorption on the glass. No changes in MY concentrations were detected in any of the LEC-free controls; therefore, the MY dye removal observed in the biosorption experiments with the biosorbent was exclusively caused by LECs.

During the MY biosorption experiments, samples were taken at different contact times between LECs and MY solutions, which were then centrifuged at 5000 rpm for 5 min. The supernatants were analyzed using visible spectrophotometry (Evolution 201 UV-Vis spectrophotometer; Thermo Fisher Scientific, Waltham, MA, USA) at 434 nm to determine their MY concentration [12].

The time-dependent MY biosorption capacity was calculated using Equation (1) [12]:

(1) $$q_{t}=\frac{\left(C_{0}-C_{t}\right)}{X}$$

where *q_t_* is the biosorption capacity (mg g^−1^) at time *t = t* (h); *C*_0_ and *C_t_* are the MY concentrations at times *t* = 0 h and *t = t*, respectively; and X is the LEC concentration (g L^−1^).

### 3.4. MY Equilibrium Biosorption Studies

MY equilibrium biosorption studies were conducted in 500 mL Erlenmeyer flasks containing 125 mL of MY solution at the previously selected pH and LEC particle size, with various initial MY concentrations (10, 20, 30, 40, 50, 60, 70, 80, 90, 100, 200, 300, 400, and 500 mg L^−1^), at 21 ± 1 °C. The flasks were shaken continuously on an orbital shaker (Cole Palmer) at 140 rpm for 72 h to ensure that a dynamic equilibrium state was reached. Samples were then collected from each of the flasks, which were centrifuged at 5000 rpm for 5 min, and the MY concentration of the supernatants was determined. The equilibrium biosorption capacity of MY (q_e_, mg g^−1^) was estimated using Equation (1); however, the C_t_ concentration was replaced by the MY equilibrium concentration in the aqueous solution (C_e_, mg L^−1^).

### 3.5. Thermodynamic Study and Mathematical Modeling of MY Biosorption Kinetics and Equilibrium

The MY biosorption kinetics at different pH levels, LEC particle sizes, initial MY concentrations, and temperatures were analyzed by four widely used two-parameter kinetic models: pseudo-first-order, pseudo-second-order, Elovich, and fractional power models (Table 4). The experimental MY equilibrium biosorption data were evaluated using two-parameter (Langmuir, Freundlich, Halsey, Temkin, and Dubinin–Radushkevich) and three-parameter (Sips, Redlich–Peterson, Radke–Prausnitz, and Toth) isotherm models (Table 4).

The E_A_ and relevant thermodynamic parameters (changes in activation entropy, enthalpy, and Gibbs free energy) of MY biosorption on LEC were calculated using the Arrhenius, Eyring–Polanyi, and Gibbs free energy models (Table 4).

### 3.6. Data and Statistical Analysis

All biosorption experiments were independently repeated thrice, ensuring reproducible and accurate results, followed by statistical data analysis. The result values are expressed as the mean value of triplicate determinations ± the standard deviation.

GraphPad Prism version 10.2.2 software (GraphPad Software, Inc., Boston, MA, USA) was employed to perform the statistical analysis of the MY biosorption data and determine the biosorption model parameters by nonlinear regression analysis.

A two-way analysis of variance was performed along with a Tukey’s test at a 5% significance level (*p* = 0.05) to determine significant differences between the means of the data groups being compared at the different levels of the evaluated batch processing factors. The different biosorption models used for fitting were assessed using the coefficient of determination (R^2^), sum of squares error (SSE), and root-mean-squared error (RMSE). A high value of R^2^ and small SSE and RMSE values correspond to a better representation of experimental data by a particular model.

## 4. Conclusions

This study investigated the effects of different physicochemical process factors on MY biosorption onto LECs, as well as the kinetics, equilibrium, and thermodynamics of biosorption. MY biosorption depended on the solution pH, LEC particle size, initial MY concentration, and temperature. The optimal pH for MY biosorption was 1.5–2.0. The highest MY biosorption rates were obtained with the smaller LEC particles (0.15–0.3 mm) and the highest temperature (62 °C). However, the MY equilibrium biosorption capacity was unaffected by the level of these process factors. Furthermore, the MY biosorption capacity increased significantly with the initial MY concentration. The pseudo-second-order model adequately described the biosorption kinetics, whereas the Sips and Redlich–Peterson models suitably represented the experimental isotherm of MY by LECs. MY biosorption by LEC is endothermic and not spontaneous. The experimental maximum LEC biosorption capacity of MY was 163.5 mg g^−1^, higher than that of most adsorbents/biosorbents. These findings indicate that LECs are a green biosorbent, highly available in nature, and strongly efficient in MY biosorption. Therefore, LECs have remarkable potential for bioremediating MY-contaminated wastewater.

## Figures and Tables

**Table 1:** Comparison of experimental results for MY biosorption on various biosorbents/adsorbents.

Biosorbent	Optimal pH	q_max_ (mg g^−1^)	Kinetic Model	Equilibrium Model	Thermodynamic Nature	Reference
Cross-linked magnetic chitosan nanoparticles	4.0	625 ^L^	PSO	Langmuir	-	[44]
Shrimp shell (*Metapenaeus monoceros*) waste	5.0	69.307 ^E^	PSO	Langmuir	Endothermic and non-spontaneous	[8]
Immobilized aquatic weed	6.0	9.9108 ^L^	PFO	Langmuir	Endothermic and spontaneous	[45]
Egg membrane	3.0	158.73 ^L^	PSO	Flory–Huggins	Endothermic and spontaneous	[7]
Spent *Rhizopus arrhizus* biomass	6.0	128.5 ^L^	PSO	Langmuir	-	[43]
Chitosan from shrimp shell (*Metapenaeus monoceros*)	4.0	199.98 ^E^	PSO	Langmuir	Exothermic and spontaneous	[15]
Bottom ash	2.0	4.77 ^L^	PFO	Langmuir Freundlich	Exothermic and spontaneous	[46]
De-oiled soya	2.0	4.02 ^L^	PFO	Langmuir Freundlich	Endothermic and spontaneous	[46]
Polyaniline–bentonite composite	7.0	444.4 ^L^	PSO	Langmuir	Endothermic and spontaneous	[47]
Pitaya fruit (*Hylocereus undatus*) peel-activated carbon	7.9	144.07 ^L^	HSDM	Langmuir	Exothermic and spontaneous	[1]
Ice-templated graphene oxide/chitosan aerogel	6.8	558.18 ^L^	PFO PSO	Langmuir	Exothermic and spontaneous	[13]
Hexadecyltrimethylammonium bromide surfactant-supported silica material	4.0	125 ^L^	PSO	Langmuir	Endothermic and spontaneous	[48]
Poplar sawdust	-	1.34 ^L^	PSO	Langmuir	Endothermic and spontaneous	[49]
Water hyacinth leaves	2.0	163.5 ^E^	PSO	Sips	Endothermic and non-spontaneous	This study

PFO, pseudo-first-order model; PSO, pseudo-second-order model; HSDM, homogeneous surface diffusion model; superscripted “^L^”, the q_max_ value was obtained from the Langmuir model; superscripted “^E^”, the q_max_ value was obtained experimentally.

**Table 2:** Parameters of the isotherm models for metanil yellow biosorption onto water hyacinth leaves.

Langmuir		Freundlich		Temkin	
q_maxL_ (mg g^−1^)	175.1 ± 5.274	K_F_ ((mg g^−1^) (mg L^−1)−1/nF^))	17.84 ± 2.769	A_T_ (L g^−1^)	0.385 ± 0.027
K_L_ (L mg^−1^)	0.032 ± 0.003	n_F_ (dimensionless)	2.468 ± 0.196	B_T_ (J mol^−1^)	31.07 ± 0.889
R^2^	0.988	R^2^	0.95	R^2^	0.990
SSE	495.5	SSE	2001	SSE	342.7
RMSE	6.174	RMSE	12.41	RMSE	5.344
Halsey		Dubinin–Radushkevich		Sips	
K_H_ (L g^−1^)	0.0009 ± 0.0008	q_maxDR_ (mg g^−1^)	141.1 ± 9.290	q_maxS_ (mg g^−1^)	170.8 ± 8.142
n_H_ (dimensionless)	−0.409 ± 0.030	B_DR_ × 10^−5^ (mol^2^ kJ^−2^)	0.0002 ± 3.04	K_S_ (L^1/ns^ mg^−1^/^ns^)	0.029 ± 0.006
R^2^	0.954	R^2^	0.883	n_S_ (dimensionless)	1.058
SSE	1627	SSE	4128	R^2^	0.992
RMSE	11.64	RMSE	18.55	SSE	316.3
				RMSE	5.134
Redlich–Peterson		Radke–Prausnitz		Toth	
K_RP_ (L g^−1^)	5.535 ± 0.576	A_R_ (L g^−1^)	6.440 ± 2.369	q_maxT_ (mg g^−1^)	192 ± 24.69
α_RP_ (L mg^−1^) ^βRP^	0.030 ± 0.013	R_R_ (L mg^−1^)	108.8 ± 100.6	B_T_ (L mg^−1^)^-nT^	0.035 ± 0.005
β_RP_ (dimensionless)	1.012 ± 0.066	B_R_ (dimensionless)	0.0868 ± 0.127	n_T_ (dimensionless)	1.235 ± 0.308
R^2^	0.992	R^2^	0.988	R^2^	0.988
SSE	324.7	SSE	437.5	SSE	462.4
RMSE	5.202	RMSE	6.306	RMSE	6.208

q_maxL_: Maximum biosorption capacity predicted by the Langmuir model; K_L_: affinity constant of the Langmuir model; K_F_: constant of the Freundlich model; n_F_: constant of the Freundlich model related to biosorption intensity; A_T_: constant of the Temkin model; B_T_: constant of the Temkin model related to sorption heat; K_H_: Halsey model constant; n_H_: Halsey model exponent; q_maxDR_: maximum biosorption capacity predicted by the Dubinin–Radushkevich model; B_DR_: constant of the Dubinin–Radushkevich model related to biosorption energy; q_maxS_: maximum biosorption capacity predicted by the Sips model; K_S_: affinity constant of the Sips model; n_s_: constant of the Sips model related to heterogeneity; K_RP_ and α_RP_: Redlich–Peterson model constants; β_RP_: Redlich–Peterson model exponent; A_R_ and R_R_: Radke–Prausnitz model constants; B_R_: Radke–Prausnitz model exponent; q_maxT_: maximum biosorption capacity predicted by the Toth model; B_T_: Toth model constant; n_T_: Toth model exponent.

**Table 3:** Thermodynamic parameters for metanil yellow biosorption onto water hyacinth leaves at different initial dye concentrations.

C_o_ (mg L^−1^)	T (°C)	ΔG (kJ mol^−1^)	E_A_ (kJ mol^−1^)	A_0_ (kJ mol^−1^ h^−1^)	ΔS (kJ mol^−1^ K^−1^)	ΔH (kJ mol^−1^)
30	21	78.20	24.30	1634	−0.1920	21.72
	35	80.88				
	50	83.76				
	62	86.07				
50	21	79.33	19.95	174.8	−0.2106	17.38
	35	82.28				
	50	85.44				
	62	87.96				
100	21	82.35	18.90	32.99	−0.2247	16.25
	35	85.49				
	50	88.86				
	62	91.56				
200	21	83.19	16.50	8.825	−0.2357	13.86
	35	86.49				
	50	90.03				
	62	92.85				

A_0_, frequency factor constant; E_A_, activation energy.

**Table 4:** Kinetic, isotherm, and thermodynamic models.

Kinetic Models			
Pseudo-first-order	${ln(q_{e1}-q}_{t})=lnq_{e1}-k_{1}t$	k_1_: Biosorption rate constant (h^−1^) q_e1_: Biosorption capacity at equilibrium (mg g^−1^)	[71]
Pseudo-second-order	$q_{t}=\frac{t}{\frac{1}{k_{2}q_{e2}^{2}}+\frac{t}{q_{e2}}}$	k_2_: Biosorption rate constant (g mg^−1^ h^−1^) q_e2_: Biosorption capacity at equilibrium (mg g^−1^)	[72]
Elovich	$q_{t}=\frac{1}{\beta_{e}}\ln\left(\alpha_{e}\beta_{e}\right)+\frac{1}{\beta_{e}}\ln t$	α_e_: Initial biosorption rate (mg g^−1^ h^−1^) β_e_: Desorption constant (mg g^−1^)	[13]
Fractional power	$q_{t}=k_{fp}\;t^{v}$	k_fp_: Model constant (mg g^−1^) ν: Rate constant (h^−1^)	[73]
Isotherm models			
Langmuir	$q_{e}=q_{maxL}\frac{K_{L}C_{e}}{1+K_{L}C_{e}}$	q_maxL_: Maximum biosorption capacity (mg g^−1^) K_L_: Affinity constant (L mg^−1^)	[62]
Freundlich	$q_{e}=K_{F}{C_{e}}^{1/n_{F}}$	K_F_: Freundlich model constant ((mg g^−1^) (mg L^−1)−1/nF^) n_F_: Constant related to biosorption intensity (dimensionless)	[74]
Temkin	$q_{e}=\frac{RT}{B_{T}}ln(A_{T}C_{e})$	A_T_: Model constant (L g^−1^) B_T_: Constant related to sorption heat (J mol^−1^) R: Ideal gas constant (8.314 J mol^−1^ K^−1^) T: Absolute temperature (K)	[75]
Halsey	$q_{e}=({\frac{K_{H}}{C_{e}})}^{\frac{1}{n_{H}}}$	K_H_: Halsey constant (L g^−1^) n_H_: Model exponent (dimensionless)	[72]
Dubinin–Radushkevich	$q_{e}=q_{maxDR}e^{-B_{DR}\varepsilon_{DR}^{2}}$ $\varepsilon_{DR}=RTln(1+\frac{1}{C_{e}})$	B_DR_: Constant related to biosorption energy (mol^2^ kJ^−2^) ε_DR_: Polanyi potential (kJ mol^−1^) q_maxDR_: Maximum biosorption capacity (mg g^−1^)	[71]
Sips	qe=qmaxSKSCe1nS1+KSCe1nS	K_S_: Affinity constant (L^1/ns^ mg^−1^/^ns^) n_s_: Constant related to heterogeneity q_maxS_: Maximum biosorption capacity (mg g^−1^)	[72]
Redlich–Peterson	$q_{e}=\frac{K_{RP}C_{e}}{1+\alpha_{RP}C_{e}^{\beta_{RP}}}$	K_RP_: Model constant (L g^−1^) α_RP_: Model constant (L mg^−1^) ^βRP^ β_RP_: Model exponent (dimensionless)	[62]
Radke–Prausnitz	$q_{e}=\frac{A_{R}R_{R}C_{e}^{B_{R}}}{A_{R}+R_{R}C_{e}^{B_{R}-1}}$	A_R_: Model constant (L g^−1^) R_R_: Model constant (L mg^−1^) B_R_: Model exponent (dimensionless)	[72]
Toth	qe=qmaxTBTCe[1+(BTCe)1nT]nT	B_T_: Model constant (L mg^−1^)^-nT^ n_T_: Model exponent (dimensionless) q_maxT_: Maximum biosorption capacity (mg g^−1^)	[74]
Thermodynamic models			
Arrhenius	$k=A_{0}{\;e}^{-\frac{E_{A}}{RT}}$	E_A_: Arrhenius activation energy (kJ mol^−1^) A_0_: Frequency factor (g mg^−1^ h^−1^)	[56]
Eyring–Polanyi	$k=\frac{k_{B}T}{h_{p}}e^{\frac{\Delta S}{R}-\frac{\Delta H}{RT}}$	∆H: Activation enthalpy change (kJ mol^−1^) ∆S: Activation entropy change (kJ mol^−1^ K^−1^) k_B_: Boltzmann constant (1.3807 × 10^−23^ J K^−1^) h_p_: Planck constant (6.6261 × 10^−34^ J s)	[56]
Gibbs	ΔG=ΔH−TΔS	∆G: Gibbs free energy change (kJ mol^−1^)	[56]

## Data Availability

All relevant data are within the paper.

## Supplementary Materials

The following supporting information can be downloaded at: https://www.mdpi.com/article/10.3390/molecules29143409/s1, Table S1: Kinetic parameters of the models for metanil yellow biosorption onto water hyacinth leaves at different solution pH values; Table S2: Kinetic parameters of the models for metanil yellow biosorption onto water hyacinth leaves at different biosorbent particle sizes; Table S3: Kinetic parameters of the models for metanil yellow biosorption onto water hyacinth leaves at different initial dye concentrations; Table S4. Kinetic parameters of the models for metanil yellow biosorption onto water hyacinth leaves at different temperatures and at four initial MY concentrations.
