# Supplementary material for: Water Hyacinth Leaves Are an Efficient, Green, and Cost-Effective Biosorbent for the Removal of Metanil Yellow from Aqueous Solution: Kinetics, Isotherm, and Thermodynamic Studies
Source: Molecules. 2024 Jul 20;29(14):3409. doi: 10.3390/molecules29143409 (PMC11279414; doi:10.3390/molecules29143409)
Supplement: Supplementary file 1 [file molecules-29-03409-s001.zip › molecules-3084851-supplementary.pdf]

Supplementary Material

Water hyacinth leaves as an efficient, green, and cost-effective biosorbent for metanil yellow removal from aqueous solution: kinetics, isotherm, and thermodynamic studies

Table S1. Kinetic parameters of the models for metanil yellow biosorption onto water hyacinth leaves at different solution pH values

		Pseudo-first-order					Pseudo-second-order				
**pH**	**$q_{e\ exp}$ (mg g^{-1})**	**q_{e1} (mg g^{-1})**	**k_1 (h^{-1})**	**R^2**	**SSE**	**RMSE**	**q_{e2} (mg g^{-1})**	**k_2 (g mg^{-1} h^{-1})**	**R^2**	**SSE**	**RMSE**
**1.5**	45.65 ± 0.619	44.13 ± 0.662	2.067 ± 0.164	0.881	1772	4.648	46.55 ± 0.576	0.073 ± 0.006	0.937	941.1	3.388
**2**	45.80 ± 0.394	44.70 ± 0.599	1.840 ± 0.127	0.915	1410	4.147	47.19 ± 0.497	0.074 ± 0.005	0.959	672.9	2.865
**3**	40.89 ± 0.650	40.61 ± 0.755	0.900 ± 0.074	0.873	1819	4.710	44.01 ± 0.839	0.030 ± 0.003	0.908	1326	4.022
**4**	33.91 ± 0.627	35.82 ± 0.286	0.368 ± 0.009	0.992	138.2	1.298	40.54 ± 0.614	0.011 ± 0.001	0.978	359.8	2.095
**5**	19.31 ± 0.548	20.65 ± 0.155	0.284 ± 0.006	0.994	32.17	0.626	23.24 ± 0.246	0.016 ± 0.001	0.990	49.19	0.775
**6**	9.16 ± 0.314	9.45 ± 0.173	0.975 ± 0.080	0.877	98.89	1.098	10.21 ± 0.173	0.014 ± 0.014	0.927	59.10	0.849
**7**	4.21 ± 0.223	4.30 ± 0.108	2.800 ± 0.405	0.703	50.38	0.784	4.549 ± 0.112	0.009 ± 0.173	0.773	38.44	0.685
		**Elovich**					**Fractional power**				
**pH**		**α_e (mg g^{-1} h^{-1})**	**β_e (g mg^{-1})**	**R^2**	**SSE**	**RMSE**	**k_{fp} (mg g^{-1})**	**υ (h^{-1})**	**R^2**	**SSE**	**RMSE**
**1.5**		2906 ± 637.3	0.194 ± 0.007	0.918	796.1	3.195	32.06 ± 0.587	0.133 ± 0.007	0.845	1507	4.396
**2**		1917 ± 453.9	0.182 ± 0.007	0.893	1218	3.952	31.73 ± 0.732	0.138 ± 0.009	0.796	2327	5.462
**3**		813.8 ± 195.1	0.191 ± 0.009	0.862	1484	4.362	25.45 ± 0.661	0.169 ± 0.010	0.828	1847	4.866
**4**		84.68 ± 11.99	0.163 ± 0.007	0.867	1941	4.989	15.51 ± 0.884	0.259 ± 0.020	0.776	3275	6.480
**5**		40.21 ± 4.65	0.287 ± 0.011	0.900	455.3	2.416	8.082 ± 0.433	0.279 ± 0.018	0.825	796.1	3.195
**6**		150.8 ± 25.06	0.775 ± 0.026	0.921	47.83	0.783	5.911 ± 0.136	0.175 ± 0.009	0.872	77.72	0.998
**7**		205 ± 57.23	1.825 ± 0.084	0.860	16.47	0.459	3.102 ± 0.061	0.156 ± 0.008	0.863	16.07	0.454

Table S2. Kinetic parameters of the models for metanil yellow biosorption onto water hyacinth leaves at different biosorbent particle sizes

Particle size (mm)		Pseudo-first-order					Pseudo-second-order				
	$q_{e\ exp}$ (mg g^{-1})	q_{e1} (mg g^{-1})	k_1 (h^{-1})	R^2	SSE	RMSE	q_{e2} (mg g^{-1})	k_2 (g mg^{-1} h^{-1})	R^2	SSE	RMSE
**0.15-0.3**	45.617 ± 0.368	43.78 ± 0.631	2.454 ± 0.183	0.909	1275	4.151	46.13 ± 0.474	0.089 ± 0.006	0.963	511.1	2.628
**0.3-0.5**	45.354 ± 0.318	41.49 ± 0.509	1.644 ± 0.094	0.952	743.2	3.169	44.52 ± 0.307	0.055 ± 0.002	0.989	176.0	1.542
**0.5-0.8**	45.950 ± 0.100	41.95 ± 0.678	0.938 ± 0.062	0.931	1073	3.809	45.71 ± 0.569	0.029 ± 0.002	0.971	458.4	2.489
**0.8-1.0**	45.599 ± 0.100	41.21 ± 0.861	0.582 ± 0.044	0.914	1289	4.174	45.53 ± 0.736	0.019 ± 0.001	0.961	584.3	2.810
**1.0-1.18**	44.113 ± 0.376	40.17 ± 0.716	0.565 ± 0.036	0.942	870.4	3.430	44.57 ± 0.576	0.018 ± 0.001	0.977	345.2	2.160
**1.18-1.4**	44.894 ± 0.343	40.76 ± 0.880	0.605 ± 0.048	0.903	1386	4.328	44.82 ± 0.796	0.017 ± 0.002	0.951	707.1	3.091
**1.4-1.7**	44.274 ± 0.221	41.59 ± 1.045	0.317 ± 0.023	0.915	1199	4.025	45.09 ± 0.875	0.011 ± 0.001	0.957	600.6	2.849
**1.7-2.0**	44.805 ± 0.345	43.23 ± 0.912	0.269 ± 0.156	0.946	835.1	3.359	47.17 ± 0.853	0.008 ± 0.001	0.968	498.1	2.594
**2.0-2.38**	42.651 ± 0.279	40.98 ± 0.562	0.279 ± 0.011	0.980	323.0	2.089	45.19 ± 0.337	0.008 ± 0.0003	0.995	77.26	1.022
		**Elovich**					**Fractional power**				
		α_e (mg g^{-1} h^{-1})	β_e (g mg^{-1})	R^2	SSE	RMSE	k_{fp} (mg g^{-1})	υ (h^{-1})	R^2	SSE	RMSE
**0.15-0.3**		3730 ± 1035	0.200 ± 0.008	0.891	975.2	3.732	32.59 ± 0.699	0.123 ± 0.008	0.791	1864	5.160
**0.3-0.5**		805.3 ± 137.0	0.174 ± 0.006	0.927	834.4	3.452	27.93 ± 0.783	0.156 ± 0.011	0.802	2255	5.676
**0.5-0.8**		491.2 ± 70.75	0.167 ± 0.005	0.936	784.7	3.348	25.38 ± 0.701	0.180 ± 0.010	0.855	1770	5.028
**0.8-1.0**		291.7 ± 39.83	0.166 ± 0.005	0.929	885.5	3.557	21.92 ± 0.606	0.210 ± 0.01	0.895	1303	4.314
**1.0-1.18**		239.8 ± 31.84	0.167 ± 0.006	0.927	897.6	3.581	20.80 ± 0.655	0.215 ± 0.011	0.877	1518	4.657
**1.18-1.4**		334.7 ± 47.72	0.173 ± 0.006	0.928	830.1	3.444	22.14 ± 0.590	0.203 ± 0.01	0.893	1237	4.204
**1.4-1.7**		166.0 ± 22.01	0.167 ± 0.006	0.916	1049	3.872	17.90 ± 0.474	0.252 ± 0.009	0.936	792.3	3.364
**1.7-2.0**		133.3 ± 1954	0.161 ± 0.007	0.888	1550	4.706	16.95 ± 0.583	0.268 ± 0.011	0.913	1203	4.145
**2.0-2.38**		86.02 ± 9100	0.152 ± 0.005	0.923	1141	4.037	15.36 ± 0.694	0.284 ± 0.015	0.885	1720	4.956

Table S3. Kinetic parameters of the models for metanil yellow biosorption onto water hyacinth leaves at different initial dye concentrations

		Pseudo-first-order					Pseudo-second-order				
C_o (mg/L)	$q_{e\ exp}$ (mg g^{-1})	q_{e1} (mg g^{-1})	k_1 (h^{-1})	R^2	SSE	RMSE	q_{e2} (mg g^{-1})	k_2 (g mg^{-1} h^{-1})	R^2	SSE	RMSE
**10**	6.285 ± 0.638	8.402 ± 0.103	1.264 ± 0.068	0.968	15.58	0.532	8.94 ± 0.136	0.225 ± 0.020	0.964	17.63	0.566
**20**	15.82 ± 0.542	16.72 ± 0.195	1.181 ± 0.059	0.972	54.5	0.995	17.95 ± 0.203	0.099 ± 0.006	0.981	37.26	0.823
**30**	24.03 ± 0.772	25.42 ± 0.385	0.839 ± 0.051	0.960	182.2	1.82	27.69 ± 0.437	0.043 ± 0.004	0.969	142.2	1.608
**40**	32.15 ± 0.803	31.86 ± 0.601	0.921 ± 0.071	0.933	465.5	2.909	34.74 ± 0.550	0.038 ± 0.003	0.966	235.9	2.071
**50**	38.91 ± 0.982	39.25 ± 0.679	0.735 ± 0.049	0.951	526.2	3.093	43.04 ± 0.670	0.024 ± 0.002	0.971	308.1	2.367
**60**	47.88 ± 0.300	46.71 ± 0.789	0.746 ± 0.049	0.954	715.6	3.607	51.32 ± 0.694	0.020 ± 0.001	0.979	331.8	2.456
**70**	57.17 ± 0.499	55.63 ± 0.975	0.604 ± 0.039	0.958	946.5	4.148	61.53 ± 0.945	0.015 ± 0.001	0.976	539.4	3.132
**80**	60.99 ± 0.506	59.97 ± 0.897	0.564 ± 0.030	0.971	761.3	3.72	66.28 ± 0.850	0.014 ± 0.0007	0.984	420.4	2.765
**90**	63.94 ± 0.115	58.70 ± 1.115	0.606 ± 0.042	0.936	2228	5.487	65.24 ± 1.097	0.013 ± 0.001	0.963	1291	4.178
**100**	75.35 ± 0.348	71.12 ± 1.221	0.427 ± 0.024	0.970	1127	4.526	79.02 ± 1.081	0.012 ± 0.0004	0.985	570.5	3.221
**200**	113.7 ± 0.011	100.8 ± 2.423	0.694 ± 2.423	0.908	6455	10.83	111.9 ± 2.162	0.011 ± 0.0008	0.957	3026	7.417
**300**	141.5 ± 0.179	121.8 ± 4.891	0.785 ± 0.124	0.911	2924	13.11	134.3 ± 4.233	0.011 ± 0.001	0.961	1298	8.739
**400**	162.6 ± 0.096	143.5 ± 6.534	1.042 ± 0.198	0.857	6003	18.79	155.9 ± 6.237	0.010 ± 0.002	0.920	3368	14.07
**500**	163.5 ± 0.328	145.7 ± 5.941	1.035 ± 0.176	0.886	4948	17.06	157.4 ± 5.499	0.010 ± 0.002	0.939	2652	12.49
		**Elovich**					**Fractional power**				
**C_o (mg/L)**		**α_e (mg g^{-1} h^{-1})**	**β_e (g mg^{-1})**	**R^2**	**SSE**	**RMSE**	**k_{fp} (mg g^{-1})**	**υ (h^{-1})**	**R^2**	**SSE**	**RMSE**
**10**		148.6 ± 49.55	0.882 ± 0.059	0.813	70.71	1.166	5.472 ± 0.243	0.151 ± 0.017	0.679	121.2	1.526
**20**		242.5 ± 60.66	0.429 ± 0.022	0.876	183.7	1.879	10.63 ± 0.423	0.163 ± 0.015	0.754	364.9	2.649
**30**		239 ± 54.28	0.272 ± 0.014	0.877	451.4	2.946	14.89 ± 0.640	0.183 ± 0.016	0.777	819.3	3.969

**40**	341.4 ± 63.99	0.218 ± 0.009	0.917	453.9	2.954	19.10 ± 0.688	0.181 ± 0.013	0.827	950.1	4.274
**50**	325.5 ± 60.96	0.175 ± 0.008	0.909	786.1	3.888	22.17 ± 0.855	0.194 ± 0.014	0.832	1453	5.286
**60**	341.6 ± 55.21	0.142 ± 0.006	0.926	941.7	4.256	26.22 ± 1.030	0.197 ± 0.014	0.835	2108	6.367
**70**	332.7 ± 58.13	0.119 ± 0.005	0.908	1707	5.729	29.35 ± 1.238	0.210 ± 0.015	0.837	3023	7.625
**80**	315 ± 54.40	0.103 ± 0.005	0.905	2113	6.375	30.86 ± 1.401	0.214 ± 0.016	0.825	3870	8.626
**90**	315.6 ± 47.03	0.110 ± 0.004	0.905	2781	6.303	30.62 ± 1.179	0.216 ± 0.014	0.832	4915	8.379
**100**	263.7 ± 40.68	0.090 ± 0.004	0.910	2899	7.467	32.81 ± 1.555	0.239 ± 0.016	0.852	4751	9.559
**200**	773.2 ± 120.7	0.067 ± 0.003	0.933	3849	8.603	55.63 ± 1.827	0.205 ± 0.012	0.884	6606	11.27
**300**	1189 ± 318.9	0.057 ± 0.003	0.944	1442	9.493	70.25 ± 3.704	0.193 ± 0.019	0.892	2799	13.23
**400**	2703 ± 920.7	0.052 ± 0.003	0.933	2083	11.41	90.48 ± 4.066	0.171 ± 0.017	0.890	3428	14.64
**500**	2570 ± 855.5	0.051 ± 0.003	0.934	2138	11.56	91.63 ± 4.450	0.169 ± 0.018	0.873	4110	16.03

Table S4. Kinetic parameters of the models for metanil yellow biosorption onto water hyacinth leaves at different temperatures and at four initial MY concentrations

			**Pseudo-first-order**					**Pseudo-second-order**				
**C_o (mg L^{-1})**	**T (°C)**	**$q_{e\ exp}$ (mg g^{-1})**	**q_{e1} (mg g^{-1})**	**k_1 (h^{-1})**	**R^2**	**SSE**	**RMSE**	**q_{e2} (mg g^{-1})**	**k_2 (g mg^{-1} h^{-1})**	**R^2**	**SSE**	**RMSE**
**30**	21	24.492 ± 0.549	24.92 ± 0.401	1.076 ± 0.073	0.928	398.3	2.32	26.84 ± 0.423	0.060 ± 0.005	0.950	278.5	1.94
**30**	35	24.764 ± 0.382	24.65 ± 0.309	2.306 ± 0.147	0.934	301.3	2.018	25.96 ± 0.232	0.149 ± 0.009	0.974	119.7	1.272
**30**	50	23.287 ± 0.462	23.38 ± 0.307	2.640 ± 0.183	0.924	306.3	2.035	24.55 ± 0.230	0.185 ± 0.012	0.969	124.2	1.296
**30**	62	23.092 ± 0.561	23.08 ± 0.366	2.928 ± 0.253	0.880	447.5	2.459	24.17 ± 0.280	0.215 ± 0.018	0.948	193	1.615
**50**	21	41.833 ± 0.734	40.94 ± 0.656	1.294 ± 0.091	0.917	1145	3.933	43.83 ± 0.578	0.046 ± 0.004	0.959	573.3	2.783
**50**	35	40.279 ± 0.575	39.29 ± 0.558	1.990 ± 0.138	0.922	942.5	3.569	41.58 ± 0.431	0.078 ± 0.005	0.968	387.6	2.289
**50**	50	41.023 ± 0.642	40.06 ± 0.563	2.565 ± 0.189	0.914	1026	3.724	42.22 ± 0.382	0.102 ± 0.006	0.972	336.2	2.132
**50**	62	40.163 ± 0.523	38.98 ± 0.636	2.825 ± 0.248	0.876	1342	4.259	40.87 ± 0.489	0.122 ± 0.011	0.947	579.4	2.798
**100**	21	78.079 ± 1.214	71.51 ± 1.497	0.557 ± 0.042	0.924	3767	7.135	79.33 ± 1.471	0.009 ± 0.001	0.955	2234	5.495
**100**	35	78.630 ± 0.567	71.68 ± 1.100	1.155 ± 0.076	0.931	3088	6.460	77.47 ± 0.896	0.022 ± 0.001	0.972	1279	4.157
**100**	50	74.678 ± 0.911	71.4 ± 1.038	1.639 ± 0.039	0.922	2598	6.090	75.58 ± 0.818	0.035 ± 0.002	0.972	937	3.659
**100**	62	72.989 ± 1.283	69.53 ± 1.159	1.582 ± 0.121	0.900	3807	7.173	73.67 ± 1.020	0.036 ± 0.003	0.947	2005	5.205
**200**	21	115.331 ± 2.072	100.7 ± 2.290	0.751 ± 0.066	0.881	10873	12.12	111.8 ± 2.147	0.009 ± 0.001	0.939	5580	8.683
**200**	35	125.564 ± 1.266	109.3 ± 2.124	1.102 ± 0.090	0.890	11298	12.36	119.4 ± 1.855	0.013 ± 0.001	0.949	5211	8.392
**200**	50	113.431 ± 0.633	105.0± 1.669	1.501 ± 0.081	0.927	4631	8.377	112.4 ± 1.366	0.020 ± 0.002	0.978	1410	4.622
**200**	62	115.47 ± 1.474	104 ± 1.849	1.566 ± 0.128	0.888	9663	11.43	110.7 ± 1.590	0.023 ± 0.002	0.944	4788	8.044
			**Elovich**					**Fractional power**				
**C_o (mg L^{-1})**	**T (°C)**		**α_e (mg g^{-1} h^{-1})**	**β_e (g mg^{-1})**	**R^2**	**SSE**	**RMSE**	**k_{fp} (mg g^{-1})**	**υ (h^{-1})**	**R^2**	**SSE**	**RMSE**
**30**	21		381.5 ± 86.49	0.293 ± 0.014	0.868	563.6	2.838	15.69 ± 0.520	0.165 ± 0.012	0.770	983.5	3.748
**30**	35		1635 ± 465.2	0.346 ± 0.015	0.877	372.9	2.308	18.1 ± 0.430	0.127 ± 0.009	0.769	702.9	3.169
**30**	50		2108 ± 656.4	0.376 ± 0.018	0.868	342.1	2.211	17.54 ± 0.402	0.121 ± 0.009	0.762	618.5	2.973

**30**	62	3499 ± 1210	0.404 ± 0.019	0.862	311.6	2.110	17.75 ± 0.372	0.113 ± 0.008	0.763	536.6	2.769
**50**	21	921.2 ± 177.1	0.186 ± 0.007	0.914	866	3.517	26.97 ± 0.697	0.156 ± 0.010	0.823	1784	5.049
**50**	35	1794 ± 424.8	0.207 ± 0.008	0.901	814.6	3.411	28.06 ± 0.666	0.136 ± 0.009	0.797	1668	4.882
**50**	50	2904 ± 720.4	0.213 ± 0.008	0.906	730.3	3.230	29.79 ± 0.639	0.126 ± 0.008	0.801	1551	4.708
**50**	62	4709 ± 1470	0.233 ± 0.010	0.878	821	3.425	29.71 ± 0.620	0.117 ± 0.008	0.780	1479	4.596
**100**	21	399.8 ± 60.23	0.092 ± 0.004	0.905	3903	7.467	36.79 ± 1.323	0.217 ± 0.013	0.850	6184	9.399
**100**	35	1071 ± 164.3	0.100 ± 0.003	0.934	2264	5.687	45.41 ± 1.234	0.169 ± 0.010	0.838	5533	8.891
**100**	50	2683 ± 524.8	0.113 ± 0.014	0.873	2619	6.299	50.76 ± 1.489	0.131 ± 0.017	0.777	4606	8.354
**100**	62	2427 ± 538.2	0.115 ± 0.004	0.904	2554	6.040	47.93 ± 1.158	0.144 ± 0.009	0.813	4996	8.448
**200**	21	927.4 ± 129.3	0.069 ± 0.002	0.933	4850	8.324	57.1 ± 1.430 0	0.200 ± 0.009	0.900	7280	10.2
**200**	35	1616 ± 192.5	0.065 ± 0.002	0.959	3198	6.759	68.31 ± 1.464	0.177 ± 0.008	0.901	7739	10.51
**200**	50	3316 ± 1212	0.075 ± 0.022	0.892	4902	8.623	73.62 ± 2.589	0.136 ± 0.032	0.799	9162	11.78
**200**	62	3217 ± 573.1	0.075 ± 0.002	0.933	4041	7.598	71.15 ± 1.568	0.149 ± 0.008	0.850	9100	11.4
